# Bacterial Immunogenicity Prediction by Machine Learning Methods

**DOI:** 10.3390/vaccines8040709

**Published:** 2020-11-30

**Authors:** Ivan Dimitrov, Nevena Zaharieva, Irini Doytchinova

**Affiliations:** Faculty of Pharmacy, Medical University of Sofia, 1000 Sofia, Bulgaria; nzaharieva@ddg-pharmfac.net (N.Z.); idoytchinova@pharmfac.mu-sofia.bg (I.D.)

**Keywords:** protective immunogens, machine learning, immunogenicity prediction

## Abstract

The identification of protective immunogens is the most important and vigorous initial step in the long-lasting and expensive process of vaccine design and development. Machine learning (ML) methods are very effective in data mining and in the analysis of big data such as microbial proteomes. They are able to significantly reduce the experimental work for discovering novel vaccine candidates. Here, we applied six supervised ML methods (partial least squares-based discriminant analysis, *k* nearest neighbor (*k*NN), random forest (RF), support vector machine (SVM), random subspace method (RSM), and extreme gradient boosting) on a set of 317 known bacterial immunogens and 317 bacterial non-immunogens and derived models for immunogenicity prediction. The models were validated by internal cross-validation in 10 groups from the training set and by the external test set. All of them showed good predictive ability, but the xgboost model displays the most prominent ability to identify immunogens by recognizing 84% of the known immunogens in the test set. The combined RSM-*k*NN model was the best in the recognition of non-immunogens, identifying 92% of them in the test set. The three best performing ML models (xgboost, RSM-*k*NN, and RF) were implemented in the new version of the server VaxiJen, and the prediction of bacterial immunogens is now based on majority voting.

## 1. Introduction

Immunogenicity is the ability of a foreign biomacromolecule (protein, lipid, carbohydrate, or a combination of them) to produce a humoral and/or cell-mediated immune response in the host organism. If the immune response leads to the production of memory cells, the immunogen is assigned as a protective immunogen. Protective immunogens of pathogenic origin are perspective vaccine candidates [1]. The identification of protective immunogens is the most important and vigorous initial step in the long-lasting and expensive process of vaccine design and development. Here, the in silico methods come to the rescue and greatly reduce the time and cost of the subsequent experimental work [2].

During the last ten years, several approaches for immunogenicity prediction of whole protein antigens have been developed [3]. Most of them like NERVE [4], Vaxign [5], ANTIGENpro, Vacceed [6], Jenner-predict [7], iVAX [8], VacSol [9], and Protectome analysis [10] work as a series of filters selecting the most probable vaccine candidates such as filters that utilize subcellular localization, adhesion probability, topology, sequence similarity with human proteins, etc. 

Only a few approaches have applied machine learning (ML) methods to classify a protein as immunogen/non-immunogen. They use positive and negative training sets of proteins of bacterial origin. Such positive sets are defined as known protective immunogens collected from the literature. The negative sets mirror the positive sets including randomly selected proteins from the same species without similarity to the positives. One of these ML approaches, VaxiJen [11], presents each protein in the set as a string of z-scales [12] describing the main physicochemical properties of the building amino acid residues and converts them to a uniform vector by auto-cross covariance (ACC) [13]. The sets of vectors were analyzed by the genetic algorithm (GA) [14] to select the relevant variables, followed by partial least squares-based discriminant analysis (PLS-DA) to derive the prediction model. VaxiJen contains models for immunogenicity prediction of proteins of bacterial, viral, parasite, fungal, and tumor origin. VaxiJen is able to analyze 100 protein sequences with an average length of 360 amino acids for 5 s and currently is the fastest tool for immunogenicity prediction [15]. The other ML tool for immunogenicity prediction, developed by Woelk’s group [16,17], uses an updated VaxiJen dataset to select the 10 most relevant features, and utilizes support vector machines (SVM) to derive the prediction model. 

Recently, a good benchmarking review of in silico tools for immunogenicity prediction was published by Rappuoli’s group [15]. They compared six servers for bacterial immunogenicity prediction (NERVE, Vaxign, VaxiJen, Jenner-predict, Bowman-Heinson, and VacSol) using the proteomes of 11 bacterial species with known immunogens as a test set and found that VaxiJen performed best in terms of running time, observed bacterial protective antigens (BPAs), sensitivity and expected BPAs, but had low fold-enrichment and high fraction of potential vaccine candidates (PVCs). 

In order to improve the fold-enrichment and PVC fraction predicted by VaxiJen, in the present study, we applied a variety of ML methods on an updated set of known bacterial antigens [18]. The applied methods were PLS-DA, *k* nearest neighbor (*k*NN), random forest (RF), support vector machine (SVM), random subspace method (RSM) with *k*NN estimator, and extreme gradient boosting (xgboost). The derived ML models were validated by receiver operating characteristic (ROC) statistics on an external test set. The three best performing models were implemented in the new version of the server VaxiJen and evaluated by the test set used in the benchmarking review [15]. 

## 2. Materials and Methods 

### 2.1. Datasets

#### 2.1.1. Dataset of Immunogenic Proteins (Positive Training Set) 

PubMed was searched for papers containing data for novel immunogenic proteins tested on humans until March 2017. Corresponding protein sequences were collected fromNCBI [19] and UniProtKB [20]. The set was curated manually for multiple protein fragments, isoforms, and duplicates. In the case of multiple protein fragments from one protein, all fragments were considered. Known epitopes were presented explicitly in the dataset, even if their parent protein was also present. In cases where several isoforms existed for one protein, all isoforms were included in the dataset. The final curated dataset consisted of 317 immunogenic proteins tested on humans derived from 47 bacterial microorganisms. The dataset included the names of bacterial microorganisms, protein names, and protein sequences in FASTA format. It is freely accessible at http://www.ddg-pharmfac.net/vaxijen/dataset [18]. 

#### 2.1.2. Dataset of Non-Immunogenic Proteins (Negative Training Set)

**Figure 1:**
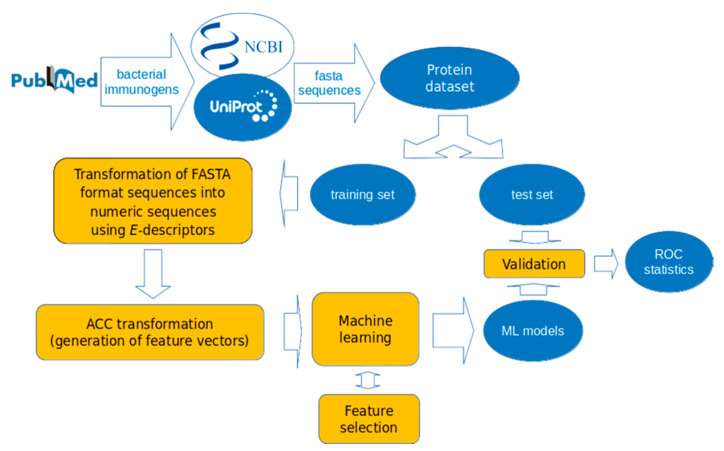
Workflow of machine learning (ML) models development in the present study. The dataset of 317 bacterial immunogens and 317 bacterial non-immunogens was divided into training and test sets. The training set was used for model development, and the test set for validation. The proteins were encoded by *E*-descriptors and auto-cross covariance (ACC)-transformed into uniform vectors. The models were validated by receiver operating characteristic (*ROC)* statistics.

A mirror dataset of 317 non-immunogenic proteins was collected from the same bacterial species as the immunogens after a BLAST search with no sequence identity to known immunogens (E-value of 0.001). The non-immunogens were selected to be similar in length to the immunogens. The collection and usage of datasets in the present study are illustrated in Figure 1.

#### 2.1.3. Training and Test Datasets

The immunogenic and non-immunogenic proteins were separated into 28 groups according to their length. Each group included between 12 and 34 proteins. Proteins in each group were scrambled and 20% of them were selected randomly as members of the test set. Thus, the training set consisted of 250 immunogenic and 250 non-immunogenic proteins and the test set contained 67 immunogenic and 67 non-immunogenic proteins. 

#### 2.1.4. Dataset for External Evaluation of the Server VaxiJen v3.0

The dataset used to evaluate the new version of VaxiJen server consisted of the proteomes of 11 bacterial species and their known bacterial protective antigens (BPA) [15]. The species were: *Neisseria gonorrhoeae, Neisseria meningitides, Staphylococcus aureus, Streptococcus pyogenes, Helicobacter pylori, Chlamydia pneumoniae, Campylobacter jejuni, Borrelia burgdorferi, Escherichia coli, Streptococcus pneumoniae*, and *Treponema pallidum*. For each species, a list of BPAs with the corresponding references is reported in Table S1. The proteomes were downloaded from the UniprotKB database [20] version 2020_04. 

### 2.2. Descriptors

The *E*-descriptors were used in the present study to quantitatively characterize the protein sequences. The *E*-descriptors were proposed by Venkatarajan and Braun [21]. They derived five numerical values for each of the 20 naturally occurring amino acids based on the principal component analysis (PCA) of 237 physicochemical properties. The first component *E1* has a strong correlation with the hydrophobicity of the amino acids. The second component *E2* gives information about the molecular size and the steric properties. The components *E3* and *E5* describe the amino acid propensity for occurrence in α-helices and β-strands, respectively. The component *E4* takes into account the partial specific volume, the number of codons, and the relative frequency of amino acids in proteins.

Each protein in our datasets was presented as a string of 5*n* elements, where *n* is the protein length. As the strings were of different length, they were transformed into uniform vectors by the auto- and cross-covariance (ACC) transformation. Dataset statistics are given in Table S2.

### 2.3. Auto-Cross Covariance (ACC) Transformation

The ACC transformation of protein sequences was introduced in 1993 by Wold et al. [13] as an alignment-independent preprocessing method for converting the different-length polypeptide chains into uniform equal-length vectors. The ACC transformation also accounts for neighbor effects. The auto- and cross-covariance are calculated by the following formulas:

(1) $$A_{jj}\left(L\right)=\overset{n-L}{\underset{i}{\sum }}\frac{E_{j,i}\times E_{j,i+L}}{n-L}$$

(2) $$C_{jk}\left(L\right)=\overset{n-L}{\underset{i}{\sum }}\frac{E_{j,i}\times E_{k,i+L}}{n-L}$$

where *j* and *k* refer to the *E*-descriptors (*j* ≠ *k*); the index *i* indicates the position of amino acid in protein (*i* = 1, 2, 3, ..., *n*); *n* is the number of amino acids in protein; and *L* is the lag (*L* = 1, 2, ..., L). Lag is the length of the frame of contiguous amino acids, for which *A_jj_* and *C_jk_* are calculated. Examples of ACC transformation are given in Table S3.

### 2.4. Machine Learning Methods

Several ML methods were applied in the present study and are described below. The WEKA software tool was used for data mining and ML [22]. The ACC-transformed sets were used as input in the models. The output from each model was 1 for the predicted antigen or 0 for the predicted non-antigen. 

#### 2.4.1. Partial Least Squares-Based Discriminant Analysis (PLS-DA)

PLS-DA uses a regression method for classification. PLS algorithm forms new attributes, named principal components (PC), as linear combinations of the initial attributes and then uses them as predictors of the dependent variable [23]. Classification procedure is based on a particular threshold. Samples are classified depending on whether they are larger or smaller than the given threshold.

#### 2.4.2. *k* Nearest Neighbor (kNN)

*k*NN measures the distances between the test data and each of the training data and classifies a data point based on how its *k* neighbors are classified [24]. We used the default WEKA parameters for the *k*NN algorithm with distance weighting equal to 1/distance. 

#### 2.4.3. Support Vector Machine (SVM)

SVM uses vectors (cases) to define a hyperplane between two classes of data by maximizing the margin between the two classes. The vectors (cases) that define the hyperplane are called support vectors. The SVM algorithm in the present study was optimized by the WEKA gridsearch algorithm. The LibSVM library [25] was used with the SVM wrapper [26]. 

#### 2.4.4. Random Forest (RF)

RF is an ensemble of individual decision trees [27]. The RF class prediction is based on the votes of individual trees. Each tree learns from a random sample of proteins and a random subset of descriptors that are randomly scrambled bootstrapping. The RF algorithm was applied in the present study with default WEKA parameters. 

#### 2.4.5. Random Subspace Method (RSM)

RSM, also known as feature bagging, reduces the correlation between estimators in an ensemble by training them on random samples of features instead of the entire feature set [28]. RF is a RSM using decision tree as an estimator. In the present study, RSM with the *k*NN algorithm as an estimator was used. RSM-*k*NN is known to be suitable for datasets with a number of features much larger than the number of training points such as gene expression data [29].

#### 2.4.6. Extreme Gradient Boosting (Xgboost)

Gradient boosting is a decision-tree-based ensemble ML algorithm proposed by Breiman [30] and later developed by other researchers [31]. Each new decision tree is grown on the weighted data from the previous one. The weights are calculated by optimizing a function indicating the fitness of model coefficients to the underlying data (loss function). The prediction of the final ensemble model is the weighted sum of the predictions made by the previous tree models. The xgboost is an advanced implementation of gradient boosting algorithm [32] and allows better control on the overfitting and gives better performance than the gradient boosting. Here, we used the WEKA wrapper for the xgboost library in R.

### 2.5. Feature Selection

Feature selection (also known as variable selection or attribute selection) is a preprocessing technique for choosing the most significant features by removing the irrelevant and redundant ones. A large number of features in one model might result in an overfitted model. The dimensionality reduction achieved through the feature selection process improves the performance of the subsequent ML algorithms [33].

Several different methods for feature selection before applying the ML algorithms were used in the present study as implemented in WEKA: correlation-based feature subset selection and consistency subset evaluation with the best first, genetic, greedy stepwise, and evolutionary search algorithm, chi-squared attribute evaluation, classifier attribute evaluation, correlation attribute evaluation, cross-validated attribute evaluation, information gain attribute evaluation, gain ratio attribute evaluation, one R attribute evaluation, and symmetrical uncertainty attribute evaluation with the ranker search algorithm.

### 2.6. Validation of the ML Models

The ML models derived in the present study were validated by internal cross-validation in 10 groups and by the external test set. The predictive ability of the models was estimated by *ROC* statistics. Four outcomes are possible in *ROC* statistics: true positives (*TP*, true immunogen predicted as immunogen); true negatives (*TN*, true non-immunogen predicted as non-immunogen); false positives (*FP*, true non-immunogen predicted as immunogen); and false negatives (*FN*, true immunogen predicted as non-immunogen). On the basis of these outcomes, four parameters were calculated: *sensitivity* (*recall*) (*TP*/total positives), *specificity* (*TN*/total negatives), *accuracy* ((*TP* + *TN*)/total) and *precision* (*TP/(TP + FP*)). These were calculated at different thresholds and the areas under the *ROC* curve (*sensitivity* vs. *1-specificity*) (*AROC*) and the *PR* curve (*precision* vs. *recall*) were estimated. *AROC* is a quantitative measure of the predictive ability and varies from 0.5 for random prediction to 1.0 for perfect prediction. The ML models were also assessed by the Matthews correlation coefficient (*MCC*), which accounts for the quality of two-class classifications [34] and by the *F1* score, which is the weighted average of *precision* and *recall*. 

### 2.7. Implementation of the Best ML Models on a Web Server

The ML models with the best performance were implemented on a web server using the Python and Django framework. The prediction of bacterial immunogens by the server is based on the majority voting of the models. 

### 2.8. Evaluation of the Server VaxiJen v.3.0

The performance of the new version of VaxiJen as a tool for bacterial vaccine antigen discovery was assessed by the dataset and the measures from Rappuoli’s benchmarking review [15]. The dataset consisted of the proteomes of 11 bacterial species. The following performance measures were considered:

- Fraction of proteome called potential vaccine candidates (PVC) (*PVCs/proteome*);
- Fraction of BPA identified within the set of PVCs (*sensitivity*).
- Fold-enrichment expressed as ratio between number of BPAs observed in the set of PVCs and the number expected drawing from the proteome a random sample of the same size of the set of PVCs (statistical significance of the fold-enrichment assessed through an hypergeometric test).

## 3. Results and Discussion

A dataset of 317 immunogenic and 317 non-immunogenic proteins from 47 bacterial species was collected as described above. They formed a training set of 250 immunogenic and 250 non-immunogenic proteins and a test set of 67 immunogenic and 67 non-immunogenic proteins. The proteins were presented as numerical strings of 5*n E*-descriptors where *n* is the number of amino acid residues. The strings of different lengths were transformed into uniform vectors by ACC-transformation with lag of 8. The lag of 8 equals the shortest peptide in the dataset. Thus, the training set was transformed into a 500 × 200 (8 × 5^2^) matrix and the test set into a 134 × 200 matrix. Examples are given in Table S3. 

Six supervised ML algorithms were applied to the training set to derive classification models for immunogenicity prediction. The performance of the derived models was assessed by 10-fold cross-validation on the training set and by prediction on the external test set. The ML methods used in the present study are described above. The workflow of model development is given in Figure 1. The performance statistics of the derived models are summarized in Table 1. 

The PLS-DA algorithm was applied on the training and test sets and showed *accuracy* 65% and 70%, respectively. This accuracy was significantly lower than the accuracy of the model in VaxiJen [11]. The RF algorithm showed better performance than PLS-DA. Different methods for feature selection were applied with the RF algorithm in attempt to improve its performance. The RF algorithm performed best with feature selection at a ranking threshold equal to 0. The ranking method reduced the number of features from 200 to 127. The feature selection gave only a slightly better prediction. In the *k*NN model, the *k* values varied between one and 10. The best performance in terms of *accuracy* and *AROC* curve was achieved at *k* = 1. The SVM model in the present study used a radial basis function for kernel type and the hyperparameters were tuned by the gridsearch algorithm. The best prediction was achieved at gamma = 100, cost = 1, and default WEKA parameters for LibSVM library. The RSM was used with the *k*NN estimator. The highest predictive ability was achieved at *k* = 1 and subspace size 0.4. The subspace size shows the size of the feature subspace used as a percentage of all of the features. The xgboost method was applied after parameter optimization. The best prediction was achieved at maxdepth = 4, eta = 1, nrounds = 150, and default parameters of xgboost package for R.

The *sensitivity* of the ML models derived in the present study ranged from 0.64 (PLS-DA) to 0.76 (*k*NN) in the 10-fold cross-validation on the training set and from 0.61 (PLS-DA) to 0.84 (xgboost) for the test set. The best performance was shown by the xgboost algorithm: 84% of the immunogens in the test set were recognized. This prediction was better distinguishable than the predictions made by the rest of the models. Apart from the PLS-DA model, all ML models performed better or equally well on the external test set than on the cross-validation in 10 groups of the training set. This is evidence for the good prediction ability without overestimation.

The *specificity* of the ML models spanned from 0.67 (PLS-DA) to 0.80 (SVM) for the training set and from 0.75 (xgboost) to 0.92 (RSM-1NN). The RSM-1NN model showed the best performance: 92% of the non-immunogens in the test set were recognized. This was followed by the *k*NN and SVM models with 84% *specificity*. Again, the predictions on the test set were better than the cross-validated predictions on the training set.

The range of the overall *accuracy* of the ML models was from 0.65 (PLS-DA) to 0.78 (RSM-1NN) for the training set and from 0.70 (PLS-DA) to 0.82 (RSM-1NN) for the test set. The highest *accuracy* of 82% on the test set was achieved by the RSM-1NN model followed by the *k*NN and xgboost models (79% *accuracy*). The *precision* of the models gave values close to their accuracies. The *AROC* was highest for the RSM-1NN model on the test set (0.88), followed by the xgboost and RF models (0.86 and 0.85, respectively). The *APR* values were similar to *AROC* ones. The *MCC* and *F1* values confirmed the good quality of the classification models. All *MCC*s were above 0. The highest *MCC* (0.66) belonged to the RSM-1NN model, while the highest *F1* (0.80) was for RSM-1NN and xgboost. 

The results showed that the xgboost model had the highest ability to recognize immunogenic proteins of bacterial origin, while the RSM-1NN model was the most powerful in the recognition of non-immunogens. Both models were derived by ensemble methods (i.e., they used combinations of classification algorithms working together). The first one used decision trees, the second used the *k*NN algorithm. The models derived in the present study better recognized the non-immunogens than the immunogens and had similar accuracies.

The three best-performing algorithms in terms of *AROC*—RSM-1NN, xgboost. and RF with feature selection—were trained on the total set of 317 bacterial immunogens and 317 non-immunogens and the derived models were implemented in the new version of VaxiJen—VaxiJen v3.0. The prediction of bacterial immunogens was based on majority voting: if two of the three models classify a given protein as an immunogen, VaxiJen returns a result “Probable ANTIGEN with probability 67%”. 

The performance of VaxiJen v3.0 as a tool for bacterial vaccine antigen discovery was evaluated by Rappuoli’s benchmarking dataset and using the same performance measures [15] as described in the Material and Methods. The majority voting for each BPA is given in Table S1. The performance measures of VaxiJen v2.0 and VaxiJen v3.0 are given in Table 2. VaxiJen v3.0 showed better performance than Vaxijen v2.0. The fraction of PVCs was only 18% of the total number of proteins with a sensitivity of 80%. The fold-enrichment increased from 1.2 for VaxiJen v2.0 to 4.5 for VaxiJen v3.0. The performances of the two versions of VaxiJen on each bacterial species are given in Table S4.

VaxiJen v3.0 is freely accessible at https://ddg-pharmfac.net/vaxijen3/. It allows an input of protein sequence in plain format (single letter code) or upload of a file with proteins in fasta format and returns probability for immunogenicity of the tested proteins. 

## 4. Conclusions

In this study, we applied six supervised ML methods on a dataset of 317 known bacterial immunogenic proteins and on a mirror dataset of non-immunogenic proteins from the same species to derive models for immunogenicity prediction. The ML models were derived after parameter optimization. The models were validated by internal cross-validation and by the external test set. All showed good predictive ability, but the most prominent ability to identify immunogens belonged to the xgboost model, while the RSM-1NN model was the best to filter the non-immunogens. The best-performing models—xgboost, RSM-1NN, and RF—were implemented in the server VaxiJen v3.0. VaxiJen v3.0 is as user friendly and comprehensive as the previous version, but shows better performance in terms of fold-enrichment and fraction of PVCs. VaxiJen is a widely used server for immunogenicity predictions with more than 500 citations (Scopus, November 2020). The current updated version of VaxiJen offers more robust predictions of bacterial immunogens while maintaining the advantages of ultra-short running time, maximum observed BPAs, highest sensitivity, and expected BPAs. 

## Figures and Tables

**Table 1:** Summary of the performance of the machine learning (ML) models. *TP*—true positives; *TN*—true negatives; *FP*—false positives; *FN*—false negatives; *AROC*—area under the *ROC* curve (*sensitivity* vs. *1-specificity*); *APR*—area under the *PR* curve (*precision* vs. *recall*), *MCC*—Matthews correlation coefficient; FS—feature selection.

Model	*TP*	*TN*	*FP*	*FN*	*Sensitivity (Recall)*	*Specificity*	*Accuracy*	*Precision*	*AROC*	*APR*	*MCC*	*F1*
**PLS-DA**												
Training set	160	168	82	90	0.64	0.67	0.65	0.66	0.70	0.66	0.31	0.65
Test set	41	53	14	26	0.61	0.79	0.70	0.74	0.74	0.76	0.41	0.67
**RF**												
*without FS*												
Training set	177	191	59	73	0.71	0.76	0.74	0.75	0.82	0.82	0.47	0.73
Test set	47	53	14	20	0.70	0.79	0.75	0.77	0.83	0.84	0.50	0.73
*with FS*												
Training set	185	190	60	65	0.74	0.76	0.75	0.76	0.82	0.82	0.50	0.75
Test set	48	55	12	19	0.72	0.82	0.77	0.80	0.85	0.83	0.54	0.76
***k*** **NN**												
Training set	191	181	69	59	0.76	0.72	0.74	0.74	0.81	0.81	0.49	0.75
Test set	50	56	11	17	0.75	0.84	0.79	0.82	0.83	0.84	0.58	0.78
**SVM**												
Training set	174	199	51	76	0.70	0.80	0.75	0.77	0.75	0.69	0.49	0.73
Test set	49	56	11	18	0.73	0.84	0.78	0.82	0.78	0.73	0.57	0.77
**RSM-1NN**												
Training set	190	198	52	60	0.76	0.79	0.78	0.78	0.85	0.87	0.55	0.77
Test set	48	62	5	19	0.72	0.92	0.82	0.91	0.88	0.89	0.66	0.80
**xgboost**												
Training set	178	179	71	72	0.71	0.72	0.71	0.72	0.79	0.80	0.43	0.71
Test set	56	50	17	11	0.84	0.75	0.79	0.77	0.86	0.88	0.58	0.80

**Table 2:** Performance measures for VaxiJen v2.0 and VaxiJen v3.0.

Performances’ Measure	VaxiJen v2.0	VaxiJen v3.0
*Number of proteins*	27,055	27,055
*PVCs*	17,256	4825
*Fraction of PVC, %*	63.78	17.83
*Sensitivity, %*	76	80
*Fold-enrichment*	1.2	4.5
*p-value*	0.00611	8.33x10^-42^

## Supplementary Materials

The following are available online at https://www.mdpi.com/2076-393X/8/4/709/s1, Table S1: List of BPAs from different species and performance of the best ML models on them; Table S2: Statistics of the training and test sets; Table S3: Examples of ACC transformation; Table S4: A detailed comparison between VaxiJen v3.0 and VaxiJen v2.0.
